# TLR4-NF-*κ*B Signal Involved in Depressive-Like Behaviors and Cytokine Expression of Frontal Cortex and Hippocampus in Stressed C57BL/6 and ob/ob Mice

**DOI:** 10.1155/2018/7254016

**Published:** 2018-03-22

**Authors:** Yihe Wang, Jingjing Xu, Yuan Liu, Ziyang Li, Xiaohong Li

**Affiliations:** ^1^School of Medicine, Shandong University, Jinan, Shandong 250012, China; ^2^Department of Medical Psychology, School of Basic Medical Sciences, Shandong University, Jinan, Shandong 250012, China; ^3^Department of Psychology, Jiangsu University Medical Center, Zhenjiang, Jiangsu 212013, China; ^4^Department of Neurology, Jinan Central Hospital Affiliated to Shandong University, Jinan, Shandong 250013, China

## Abstract

Studies found that elevated levels of cytokines such as interleukin- (IL-) 1*β*, IL-6, and tumor necrosis factor-*α* (TNF-*α*) are closely associated with the pathogenesis of depression. Obesity providing a low-grade inflammation state was proposed to be implicated in susceptibility to depression in obesity. However, the alterations of cytokines and the TLR4-NF-*κ*B signal in the brain of normal-weight and obese mice under stress have not been fully elucidated. This study used chronic unpredictable mild stress (CUMS) to induce a depressive-like behavior in an animal model and examine depressive-like behaviors, memory changes, and serum corticosterone levels, as well as the expressions of cytokines and NF-*κ*B in the frontal cortex and hippocampus. We aimed to observe the role of neuroinflammation in susceptibility to depression in obesity under CUMS. In addition, we investigated the protective effect of inhibiting the TLR4-NF-*κ*B signal. Our results demonstrated that CUMS induced depressive-like behavior and spatial memory damage, higher level of serum corticosterone, and overexpression of cytokines and NF-*κ*B in the frontal cortex and hippocampus in both C57BL/6 and ob/ob mice. ob/ob mice displayed serious behavioral disorder and higher levels of IL-1*β*, IL-6, TNF-*α*, and NF-*κ*B. Our results concluded that a hyperactive TLR4-NF-*κ*B signal and higher level of cytokines are involved in susceptibility to depression in stressed obese mice.

## 1. Introduction

An increasing amount of literatures have reported that neuroinflammation is linked with etiology of depression [1–3]. Immune activation and elevated levels of cytokines in the brain are closely associated with severity of depression [4–6]. Further, some cytokines such as interleukin- (IL-) 1*β*, IL-6, and tumor necrosis factor-*α* (TNF-*α*) were believed to be contributors of the onset and progression of depression [7–9] because these cytokines affected the neuron function and neuroactive molecule production which were associated with depression [10–12]. Meanwhile, some anti-inflammation medications could relieve symptoms of depression by reducing the overexpression of cytokines [7, 13–15].

Recently, a great body of researches has demonstrated that depression and obesity are coexisting [16–18]. The high-fat diet combined with chronic unpredictable mild stress (CUMS) could induce a serious depressive-like behavior in rats [19]. Neuroinflammation was the link of the pathophysiology of obesity comorbidity with depression as obesity provided a low-grade inflammation state [17, 18, 20]. Specially, increased damage-associated molecular pattern (DAMP), an active element of the TLR4/NF-*κ*B signal, has been found in stress reaction which was regarded as the major reason of depression [16]. Higher levels of IL-1*β*, IL-6, and TNF-*α* in the brain were believed to induce depression due to DAMPs under stress [21]. Our previous studies revealed that both long-term mild stress and lipopolysaccharide (LPS) induced depressive-like behaviors as well as IL-1*β*, IL-6, and TNF-*α* expression in the frontal cortex and hippocampus [7, 22]. These results supported the conclusion that the activation of TLR4-NF-*κ*B is involved in the pathogenesis of both depression and obesity. However, the role of the TLR/NF-*κ*B signal in susceptibility to depression of stressed obesity remains unclear.

TLR4 belongs to the TLR family, which is composed of evolutionarily conserved microbe-specific structural motifs and the endogenous molecule recognition transmembrane TLR4 expressed in various sentinel cells of the central nervous system and formed the first line of defense in the brain [23, 24]. The sustained expression of TLR4 induced neuroinflammation which constitutes the reason of psychiatric disorders [21]. LPS induced both depressive-like behavior and cytokine expression in the hippocampus of mice, and mice lacking caspase-1 resisted LPS-induced depressive-like behavior [22]. The above studies suggested that activated TLR4 might play a crucial role in the pathophysiology of depression. NF-*κ*B is a transcription factor which induces a certain cytokine expression and regulates the inflammatory cascade [25, 26]. More importantly, studies have clearly shown that NF-*κ*B increased IL-1*β* and TNF-*α* expression in the hippocampus and frontal cortex during stress [7] and regulated neurogenesis in the hippocampus which were closely related with depression [27, 28].

In the study, CUMS was used to establish an animal model for depression, which replicates the specific neuroendocrinological and cytokine expression abnormalities of depression patients [29]. In addition, ob/ob mice with higher levels of blood lipid and C57BL/6 mice were used in order to distinguish the differences in behavioral alteration and cytokine expression between normal-weight and obese mice. Given that a higher level of blood lipid is one of the DAMPs that could initiate TLR4 and inhibition of the TLR4-NF-*κ*B signaling pathway, which might be a potential strategy in depression treatment, the aim of the study was to compare the depressive-like behaviors and the levels of cytokines (IL-1*β*, IL-6, and TNF-*α*) and NF-*κ*B in the brain between C57BL/6 mice and ob/ob mice after CUMS and whether the TLR4 antagonist Tak242 could reverse the depressive-like behaviors and overexpressions of cytokines and NF-*κ*B in stressed mice.

## 2. Materials and Methods

### 2.1. Animals

Thirty male C57BL/6 mice, seven to eight weeks old, weighing 18–22 grams and thirty male ob/ob mice, seven to eight weeks old, weighing 40–45 grams obtained from Vital River Laboratory Animal Technology Co., Ltd. (Beijing, China), were housed (5 per cage) and maintained under a 12 hr light-dark cycle, at 20–24°C with free access to food and water. The weights of mice were recorded at baseline and every week during the CUMS procedure and on the day after behavioral tests. The order of behavioral tests was sucrose preference test, open-field test, and Morris water maze test. All procedures used in this study were reviewed and approved by the Ethics Committee of School of Medicine, Shandong University, which complies with the National Institutes of Health's *Guide for the Care and Use of Laboratory Animals* (NIH publication no. 85-23, revised 1985).

### 2.2. Experimental Groups and Drug Administration

After 7 days of adaptation, C57BL/6 mice and ob/ob mice were randomly divided into 6 groups (10 mice per group): C57BL/6 control group (Ctrc57), C57BL/6 CUMS group (CUMSc57), C57BL/6 CUMS + Tak242 (Takc57), ob/ob control group (Ctrob), ob/ob CUMS group (CUMSob), and ob/ob CUMS + Tak242 group (Takob). Two CUMS groups received CUMS procedure, and two Tak242 groups received both CUMS procedure and a daily intraperitoneal injection of TLR4 antagonist Tak242 (3 mg/kg, freshly suspended in 80% polyethylene glycol 400) at 30 min prior to stress exposure [30]. The control and CUMS groups were given 80% polyethylene glycol 400 (vehicle) to balance the systematic error.

### 2.3. Chronic Unpredictable Mild Stress

Mice in the CUMS and CUMS + Tak242 groups were repeatedly exposed to a series of chronic unpredictable mild stressors that included the following: 8 hours of food deprivation, 8 hours of water deprivation, 45° lean cage, white noise (1500 Hz, 92 PB, 1 h), day and night confusion, foot shock for 15 min (50 mV, 10 s duration, average 1 shock/min), and horizontal oscillation for 20 min. Each stressor was applied per day, and the entire stress procedure lasted for 3 weeks, with stressors applied in a completely random order, inducing a depressive state [31].

### 2.4. Behavior Tests

#### 2.4.1. Sucrose Preference Test

A decreased sucrose preference is considered to be homologous to anhedonia, the inability to experience pleasure, which simulates the defining symptom of major depression [32, 33]. Mice were individually housed during the sucrose preference test. Prior to the test, there was a 48-hour adaptation period for mice to habituate to sucrose solution or water. After that, a 23-hour period of water and food deprivation was carried out. Then, each mouse was given free access to two bottles for 1 hour, one with 200 ml 1% (w/v) sucrose solution and the other with 200 ml water. The drinking bottles were weighed to calculate the consumption of fluids 1 hour later. The sucrose preference percentage was evaluated by the amount of sucrose solution consumed during consumption of all fluids, the decrease of which indicated the depression symptom.

#### 2.4.2. Open-Field Test

An open-field test was used to test the exploration motivation of mice [34, 35]. The open-field apparatus is made of black acrylic (90 cm diameter and 45 cm wall height). Mice were placed individually at the center of the apparatus and left free to explore the arena for 5 min. The following indices were recorded: number of grid crossings (horizontal movement), defined as crossing into the nearby grids with more than three paws or half of the body; number of rears (vertical movement), defined as both forelimbs raised at least 1 cm above the ground; frequency of grooming behaviors; and time in centre squares, defined as the time spent in the central nine grids before stepping into the outer grids of the apparatus. The box was thoroughly cleaned between tests. The time in centre squares, horizontal and vertical movements, and number of grooming were recorded by a camera linked with a computer which fitted with a SMART video tracking system in the study (SMART v3.0, Panlab, Spain).

#### 2.4.3. Morris Water Maze

A version of the conventional Morris water maze test was performed to evaluate spatial reference learning and memory [36]. The maze was a blue pool (0.8 m in diameter) filled with water (0.3 m deep, 25 ± 1°C). Geometric pictures pasted on the surrounding walls were used by the mice for space orientation. Four equal quadrants were divided according to four directions: I, II, III, and IV. Movement tracks of the mice were captured by a CCD camera connected with a computer. All mice were allowed to swim for 60 s freely within 24 hours before the formal training. During the following 4 consecutive days, mice were trained to find a platform (12 cm diameter) hidden under the water surface in quadrant IV 4 times per day. If a mouse failed to find the platform within 60 s, it was manually guided to the platform and allowed to remain there for 10 s. The escape latency was scored as 60 s for these mice. On the fifth day, all mice were released into the maze without the hidden platform from an identical point of quadrant I and allowed to swim freely for 60 seconds. The percentage of time spent in quadrant IV of mice on the fifth day was analyzed.

### 2.5. Serum Corticosterone Measurement

At the end of the experiment, the animals (six per group) were sacrificed under deep anesthesia; all blood samples were obtained and centrifuged for 10 min at 3000 rpm. Serum corticosterone (CORT) was measured using a commercially available radioimmunoassay (RIA) kit (Nanjing Jiancheng Bioengineering Institute, China). RIA was performed according to the manufacturer's instruction.

### 2.6. Enzyme-Linked Immunosorbent Assay

Animals (six per group) were sacrificed under deep anesthesia, and both sides of the frontal and parietal bones were pulled off to collect the whole brain from the cranial cavity. After that, the frontal cortex and hippocampus were collected and immersed immediately in liquid nitrogen and stored at −80°C for further protein isolation. The tissue was dissociated using an ultrasonic cell disruptor and lysed in cold lysis buffer containing 10 mM Tris-HCl, pH 8.0, 240 mM NaCl, 5 mM EDTA, 1 mM dithiothreitol, 0.1 mM phenylmethanesulfonyl fluoride, 1% Triton X-100, 1 mM sodium vanadate, and 1 g/ml of leupeptin, pepstatin, and aprotinin. Tissue lysates were incubated at 4°C for 20 min. The sample was centrifuged at 12,000 rpm for 10 min at 4°C, then the supernatant was collected and protein content was determined by BCA protein assay reagents (Pierce, Rockford, IL). The levels of IL-1*β*, IL-6, and TNF-*α* were measured using commercially available enzyme-linked immunosorbent assay (ELISA) kits according to the manufacturer's instructions (Beijing Ke Ying Mei Technology Co., Ltd, China). Briefly, serial dilutions of protein standards and samples were added to 96-well ELISA plates, followed by biotinylated antibodies of IL-1*β*, IL-6, and TNF-*α*. After rinsing with wash buffer, a prepared solution of the avidin-horseradish peroxidase conjugated complex was added followed by addition of substrate solution. The reaction was stopped by the stopping solution. The optical density was detected at 450 nm using the iMark Microplate Absorbance Reader (Bio-Rad Labs, Hercules, CA, USA). The concentration of each sample was calculated from the linear equation derived from the standard curve of known concentrations of the cytokines.

### 2.7. Western Blotting

#### 2.7.1. Protein Isolation

Brain tissue was homogenized in a lysis buffer supplemented with 1% protease inhibitor phenylmethanesulfonyl fluoride (PMSF) in a ratio of 1 : 5 (1 g tissue/5 ml reagent). The lysed tissue sample was centrifuged at 14000*g* at 4°C for 30 min, and then the protein-containing supernatant obtained was either immediately used or stored at −80°C. The protein concentration was detected with a BCA Protein Assay Kit (Beyotime Institute of Biotechnology) using the iMark Microplate Absorbance Reader (Bio-Rad, CA, USA).

#### 2.7.2. NF-*κ*B Western Blotting

Brain protein samples containing the same amount of total protein were mixed with a 5x Laemmli loading buffer (protein volume : loading buffer = 4 : 1). The mixed protein sample was heated at 99°C for 5 min to cause protein denaturation, and then 20 *μ*g of protein sample was separated on 12% sodium dodecyl sulfate-polyacrylamide (SDS-PAGE) gel and electrotransferred to polyvinylidene difluoride (PVDF) membranes (Bio-Rad, CA, USA). The membrane was blocked with 5% skim milk in TBS containing 0.1% Tween-20 (TBST) for 1 h and incubated with primary antibodies against NF-*κ*B p65 protein (anti-rabbit, 1 : 2500, Abcam, USA) or GAPDH (anti-rabbit, 1 : 5000, Biogot Technology Co., Ltd) at 4°C overnight in a refrigerator. The following day, after washing with TBST three times for 5 min, the PVDF membrane was incubated for 1 h at room temperature with the secondary antibody (anti-rabbit, 1 : 10000, ZSGB-BIO, China). Then, the PVDF membrane was washed again with TBST three times for 15 min; the Western blots were visualized after being incubated with ECL solution (Millipore Corp., Billerica, Massachusetts, USA) for 1 min and exposed onto photographic films (Eastman Kodak Company, Rochester, New York, USA) for 10–90 sec. Signal intensities were quantified using the ImageJ 14.0 software, and the density value of the objective protein band was normalized according to that of the GAPDH band of the same sample.

### 2.8. Immunohistochemistry

The animals (four per group) were anesthetized with pentobarbital and perfused with 50 ml of 0.1 M PBS, followed by 100 ml ice-cold 4% paraformaldehyde (PFA). Paraffin-embedded frontal cortex and hippocampus tissues were cut to a thickness of the 5 *μ*m section on a microtome. After rehydration, the sections were incubated with fresh 3% H_2_O_2_ for 10 min under room temperature, washed with distilled water, and heated at 95–98°C in 0.01 M citrate buffer (pH 6.0) for 15 min, then cooled at room temperature for 30 min and washed with PBS. Then the sections were blocked with normal goat serum for 20 min, incubated with rabbit anti-NF-*κ*B p65 antibody (1 : 250, GeneTex, USA) at 4°C overnight, and washed with PBS. After that, the sections were incubated with biotinylated goat anti-rabbit IgG antibodies at 37°C for 15 min, rewashed with PBS, incubated with the streptavidin-biotin complex (SABC) at 37°C for 15 min, and then stained with diaminobenzidine under a microscope. At the end, the sections were dehydrated, cleared, and mounted. After all the staining was finished, each section was viewed under 400x magnification, and NF-*κ*B p65 expression was observed.

### 2.9. Statistics

Data were presented as the mean ± SEM. Statistical analysis of data was carried out by one-way analysis of variance (ANOVA) with S-N-K post hoc test and independent samples *t*-test. Further, two-way ANOVA was used to analyze the change in bodyweight during the CUMS procedure. Differences were considered statistically significant if the *p* value was less than 0.05.

## 3. Results

### 3.1. Body Weight

**Figure 1:**
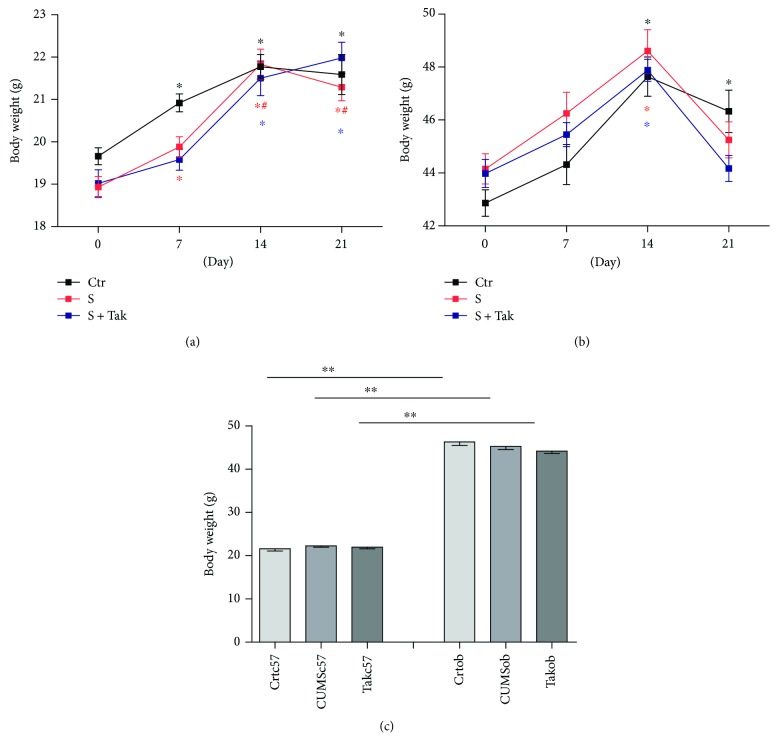
The comparison of body weight in different groups. (a) Body weight changes during CUMS of C57BL/6 mice; ^∗^*p* < 0.05 compared with 0 day and ^#^*p* < 0.05 compared with 7 days. (b) Body weight changes during CUMS of ob/ob mice; ^∗^*p* < 0.05 compared with 14 days. (c) The body weight on the day after behavioral tests. Results are expressed as mean ± SEM (*n* = 10 in each group); ^∗∗^*p* < 0.01.

The results showed that the main effect of group on body weight was not significant (*F* = 2.093, *p* = 0.128; *F* = 1.764, *p* = 0.177), but the time effect on body weight was significant in C57BL/6 groups (*F* = 42.227, *p* < 0.001) and ob/ob groups (*F* = 24.327, *p* < 0.001) during the period of CUMS (Figures 1(a) and 1(b)). The body weight of both C57BL/6 and ob/ob mice increased during 14 days of the CUMS procedure. Briefly, the body weights of the C57BL/6 control group and CUMS group significantly increased at 7 days of the CUMS procedure compared with those at baseline (*F* = 9.157, *p* < 0.001) (*p* < 0.05, resp.), and the body weight of the C57BL/6 Tak242 group significantly increased at 14 days of the CUMS procedure compared with that at baseline (*p* < 0.05, resp.). The body weights of all ob/ob groups significantly increased at 14 days of CUMS procedure compared with those at baseline (*p* < 0.05, resp.). There were significant differences in body weight between C57BL/6 mice and ob/ob mice in control groups (*t* = 27.731, *p* < 0.001), CUMS groups (*t* = 32.160, *p* < 0.001), and Tak242 groups (*t* = 36.014, *p* < 0.001) after behavioral tests (Figure 1(c)). The results indicated that there was no effect of the CUMS regimen on body weight of both C57BL/6 mice and ob/ob mice, but ob/ob mice had greater body weight than C57BL/6 mice had on the day after behavioral tests due to the animal strains.

### 3.2. Behavioral Tests

#### 3.2.1. Sucrose Preference Test

**Figure 2:**
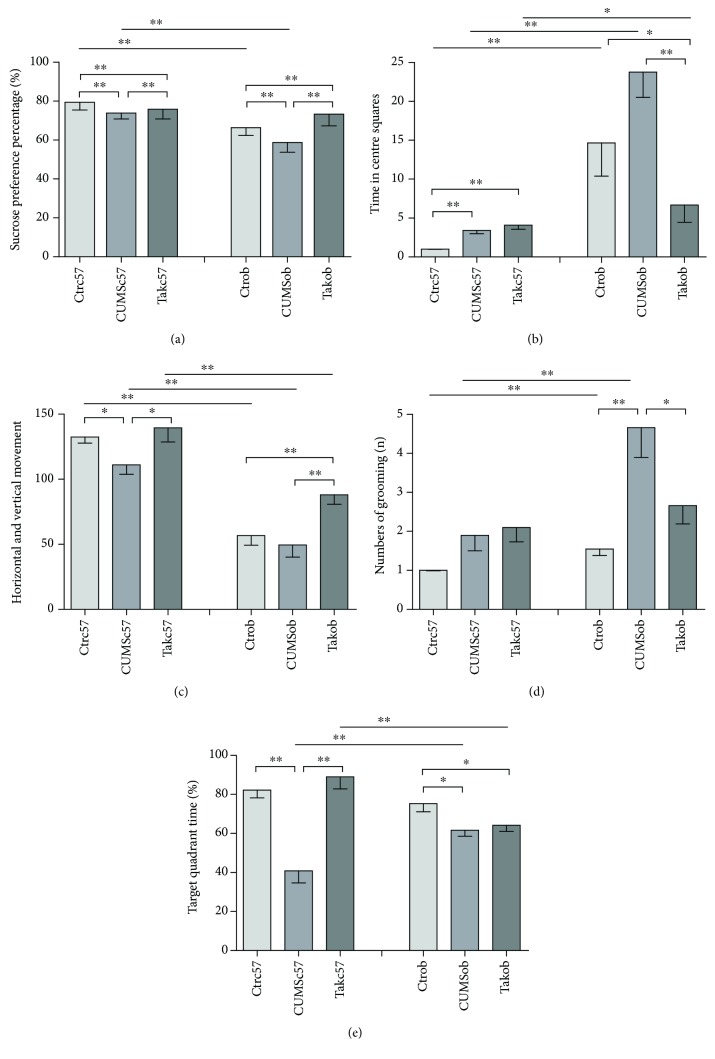
The comparison of behavior changes in different groups: (a) sucrose preference percentage in the SPT; (b) time in centre squares in the OFT; (c) number of horizontal and vertical movements in the OFT; (d) number of grooming in the OFT; (e) percentage of time spent in the target quadrant in the MWM test. Results are shown as mean ± SEM (*n* = 10 in each group). ^∗^*p* < 0.05 and ^∗∗^*p* < 0.01.

As shown in Figure 2(a), CUMS exposure significantly reduced the percentage of sucrose consumption in both stressed C57BL/6 mice and ob/ob mice in comparison with the control animals [*F* = 76.236, *p* < 0.001; *F* = 22.252, *p* < 0.001], while treatment with Tak242 significantly prevented the decrease in sucrose consumption, as compared to the CUMS-exposed mice (*p* < 0.01; *p* < 0.01). In addition, the Ctrob and CUMSob groups had lower percentage of sucrose consumption than the Ctrc57 and CUMSc57 groups had (*t* = 6.827, *p* < 0.001; *t* = 20.646, *p* < 0.001). The results indicated that stress decreased sucrose preference in C57BL/6 mice and ob/ob mice, while the antagonist reversed the alteration. Both unstressed and stressed ob/ob mice had lower sucrose consumption than C57BL/6 mice had.

#### 3.2.2. Open-Field Test

As shown in Figure 2(b), CUMS increased the time in centre squares in C57BL/6 mice [*F* = 16.483, *p* < 0.001] (*p* < 0.01), but Tak242 could not relieve the alteration (*p* < 0.01). There was a significant difference in the time in centre squares among ob/ob mice [*F* = 4.334, *p* = 0.025], unstressed and stressed mice had a longer time in centre squares compared with the antagonist group (*p* < 0.05; *p* < 0.01). Moreover, all ob/ob groups had a longer time in centre squares than their C57BL/6 counterparts had (*t* = 3.378, *p* = 0.004; *t* = 6.531, *p* < 0.001; and *t* = 2.706, *p* = 0.015). As shown in Figure 2(c), the C57BL/6 mice in the CUMS group showed decreased horizontal and vertical movements [*F* = 3.496, *p* = 0.045] in comparison to the control group (*p* < 0.05). Treatment with Tak242 significantly reversed the behavioral alteration as compared to the CUMS groups (*p* < 0.05). In ob/ob mice, unstressed and stressed mice had lower horizontal and vertical movements compared with the antagonist group [*F* = 6.463, *p* = 0.006], Tak242 prevented the lower exploratory behaviors (*p* < 0.01). In addition, all ob/ob groups had decreased horizontal and vertical movements compared with the C57BL/6 group (*t* = 8.860, *p* < 0.001; *t* = 5.268, *p* < 0.001; and *t* = 3.860, *p* = 0.001). As shown in Figure 2(d), CUMS increased the number of grooming in ob/ob mice [*F* = 8.915, *p* = 0.001] (*p* < 0.01) while Tak242 relieved the change (*p* < 0.05). The control ob/ob group and CUMS ob/ob group displayed a much higher number of grooming than did C57BL/6 groups (*t* = 3.344, *p* = 0.004, and *t* = 3.291, *p* = 0.004, resp.).

The results indicated that stress induced lower interest in a new environment. Behavior suppression of both C57BL/6 mice and ob/ob mice was explored; ob/ob mice had obvious behavioral disorders in the OFT compared with C57BL/6 mice, and the antagonist improved most of the abnormal behaviors in both mice and ob/ob mice.

#### 3.2.3. Morris Maze Test

As shown in Figure 2(e), CUMS mice showed less target quadrant time in the Morris maze test [*F* = 22.123, *p* = 0.001, and *F* = 4.283, *p* = 0.026] in comparison to the control mice (C57BL/6 mice: *p* < 0.01; ob/ob mice: *p* < 0.05). Treatment with Tak242 significantly reversed the behavioral alteration as compared to the CUMS C57BL/6 mice (*p* < 0.01), while Tak242 treatment did not improve the behavioral change in the Tak242 ob group (*p* > 0.05). Moreover, the CUMSc57 group had less target quadrant time compared with the CUMSob group (*t* = 2.889, *p* = 0.010), while Tak242 c57 had a longer target quadrant time compared with the Takob group in the Morris maze test (*t* = 3.451, *p* = 0.003). The results indicated that CUMS induced less target quadrant time in both C57BL/6 mice and ob/ob mice in the Morris maze test, and the antagonist relived the alteration in C57BL/6 mice.

### 3.3. The Level of Serum Corticosterone

**Figure 3:**
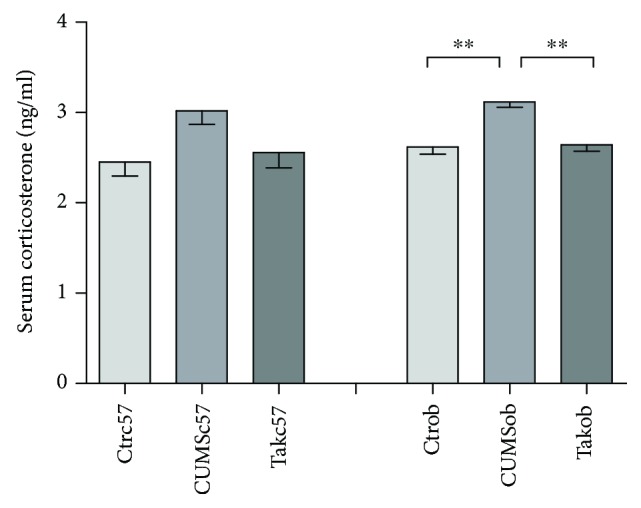
The comparison of serum corticosterone levels in different groups. Data are expressed as means ± SEM (*n* = 6 in each group). ^∗∗^*p* < 0.01.

As shown in Figure 3, CUMS induced a higher level of serum corticosterone in stressed ob/ob mice [14.070, *p* < 0.001] compared with unstressed mice (*p* < 0.01). Tak242 could reverse the increased level of corticosterone in ob/ob mice (*p* < 0.01).

### 3.4. The Levels of Cytokines in the Frontal Cortex and Hippocampus

**Figure 4:**
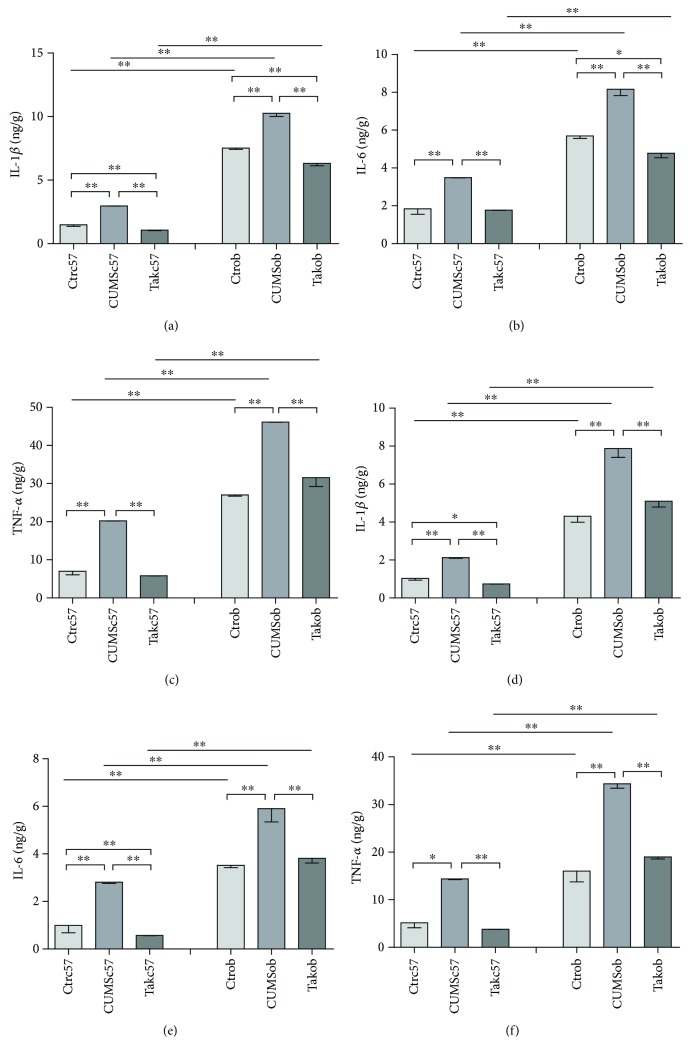
Cytokine expression in the frontal cortex and hippocampus measured in different groups: (a) IL-1*β* expression in the frontal cortex; (b) IL-6 expression in the frontal cortex; (c) TNF-*α* expression in the frontal cortex; (d) IL-1*β* expression in the hippocampus; (e) IL-6 expression in the hippocampus; (f) TNF-*α* expression in the hippocampus. Data are expressed as means ± SEM (*n* = 6 in each group). ^∗^*p* < 0.05 and ^∗∗^*p* < 0.01.

Cytokine expression in the frontal cortex is shown in Figures 4(a)–4(c). As shown in Figure 4(a), the level of IL-1*β* increased in both stressed C57BL/6 mice [*F* = 244.847, *p* < 0.001] (*p* < 0.01) and ob/ob mice as compared to controls [*F* = 251.203, *p* < 0.001] (*p* = 0.01), while Tak242 could reverse the alteration in C57BL/6 mice and ob/ob mice (*p* < 0.01; *p* < 0.01). ob/ob mice in the control group, CUMS group, and Tak242 group had higher levels of IL-1*β* than C57BL/6 mice had (*t* = 39.963, *p* < 0.001; *t* = 298.468, *p* < 0.001; and *t* = 27.188, *p* < 0.001). As shown in Figure 4(b), CUMS significantly increased the levels of IL-6 in C57BL/6 mice [*F* = 32.093, *p* = 0.001] (*p* < 0.01) and ob/ob mice [*F* = 67.729, *p* < 0.001] (*p* < 0.01) as compared to controls, while Tak242 decreased the levels of IL-6 in C57BL/6 mice (*p* < 0.01) and ob/ob mice (*p* < 0.01). Further, ob/ob mice in the control group, CUMS group, and Tak242 group had higher levels of IL-6 than C57BL/6 mice had (*t* = 11.741, *p* < 0.001; *t* = 13.777, *p* < 0.001; and *t* = 82.866, *p* < 0.001). As shown in Figure 4(c), stress significantly increased TNF-*α* in C57BL/6 mice [*F* = 215.849, *p* < 0.001] (*p* < 0.01) and ob/ob mice [*F* = 54.251, *p* < 0.001] (*p* < 0.01), while Tak242 could reverse the change in TNF-*α* level (*p* < 0.01; *p* < 0.01). The Ctrob, CUMSob, and Tak242 ob groups had higher levels of TNF-*α* than C57BL/6 groups had, respectively (*t* = 20.156, *p* < 0.001; *t* = 353.631, *p* < 0.001; and *t* = 11.044, *p* < 0.001).

The results indicated that CUMS induced increased IL-1*β*, IL-6, and TNF-*α* levels in the frontal cortex in both C57BL/6 and ob/ob mice, while the antagonist reversed the alteration; ob/ob mice had higher levels of cytokines in the frontal cortex than C57BL/6 mice had.

Cytokine expression in the hippocampus is shown in Figures 4(d)–4(f). The change tendency was similar to the frontal cortex.  Figure 4(d) shows that the levels of IL-1*β* increased in both CUMS C57BL/6 group [*F* = 167.546, *p* < 0.001] (*p* < 0.01) and CUMS ob/ob group compared to the control group [*F* = 24.858, *p* = 0.001] (*p* < 0.01); however, Tak242 reversed the alteration in C57BL/6 mice and ob/ob mice (*p* < 0.01; *p* < 0.01). Unstressed, stressed, and Tak242 ob/ob mice had higher levels of IL-1*β* than C57BL/6 mice had (*t* = 10.107, *p* = 0.001; *t* = 12.004, *p* < 0.001; and *t* = 13.796, *p* < 0.001).  Figure 4(e) shows that CUMS significantly increased the levels of IL-6 in C57BL/6 [*F* = 41.508, *p* < 0.001] (*p* < 0.01) and ob/ob mice [*F* = 13.651, *p* = 0.006] (*p* < 0.01) compared to the control, while Tak242 decreased the levels of IL-6 in C57BL/6 mice (*p* < 0.01) and ob/ob mice (*p* < 0.01). Meanwhile, unstressed, stressed, and Tak242 ob/ob mice had higher levels of IL-6 than C57BL/6 mice had (*t* = 7.676, *p* = 0.002; *t* = 5.443, *p* = 0.006; and *t* = 15.702, *p* < 0.001).  Figure 4(f) shows that stress increased TNF-*α* in C57BL/6 [*F* = 94.544, *p* < 0.001] (*p* < 0.05) and ob/ob mice [*F* = 47.300, *p* < 0.001] (*p* < 0.01), while Tak242 could reverse the change in TNF-*α* level (*p* < 0.01; *p* < 0.01). ob/ob mice in the control, CUMS, and antagonist groups had higher levels of TNF-*α* than C57BL/6 groups had (*t* = 4.381, *p* = 0.012; *t* = 21.203, *p* < 0.001; and *t* = 37.023, *p* < 0.001).

The results suggested that CUMS induced increased IL-1*β*, IL-6, and TNF-*α* levels in the hippocampus in C57BL/6 mice and ob/ob mice, while the antagonist reversed the alteration; ob/ob mice in unstressed, stressed, and antagonist groups had higher levels of cytokines in the hippocampus than C57BL/6 mice had.

### 3.5. NF-*κ*B p65 Expression in the Frontal Cortex and Hippocampus

#### 3.5.1. NF-*κ*B p65 Expression in the Frontal Cortex and Hippocampus in Western Blot

**Figure 5:**
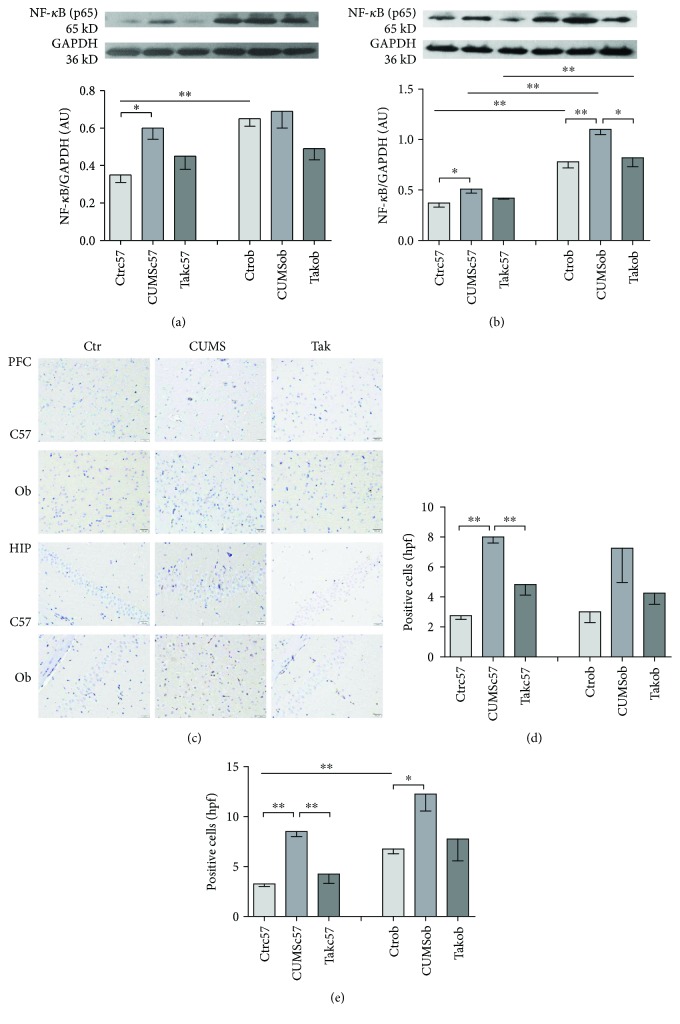
NF-*κ*B p65 expression in the frontal cortex and hippocampus. (a) NF-*κ*B p65 expression in the frontal cortex measured in Western blot. (b) NF-*κ*B p65 expression in the hippocampus measured in Western blot. (c) NF-*κ*B p65 expression in the frontal cortex and hippocampus in immunohistochemistry (×400). Above: frontal cortex; below: hippocampus. (d) Positive expression neuron counting in the frontal cortex in a high-power field (hpf). (e) Positive expression neuron counting in the hippocampus in a high-power field (hpf). Data are expressed as means ± SEM (*n* = 6 in each group and *n* = 4 in each group). ^∗^*p* < 0.05 and ^∗∗^*p* < 0.01.

Figure 5(a) shows the NF-*κ*B p65 expression in the frontal cortex in Western blot. The CUMS procedure induced a marked increase in NF-*κ*B p65 level in C57BL/6 mice compared to control animals [*F* = 4.043, *p* = 0.035] (*p* < 0.05), and Tak242 could relieve the alteration (*p* > 0.05 control group versus Tak242 group). CUMS did not induce a significant increase in NF-*κ*B p65 level in ob/ob mice [*F* = 2.359, *p* = 0.123]. Moreover, the NF-*κ*B p65 level of ob/ob mice in the control group was higher than that of the C57BL/6 mice in the control group (*t* = 4.669, *p* = 0.001). The results suggested that both unstressed and stressed ob/ob mice had higher levels of NF-*κ*B p65 than C57BL/6 mice in the frontal cortex, but the antagonist could not reverse the alteration of the NF-*κ*B p65 level in ob/ob mice.

Figure 5(b) shows the NF-*κ*B p65 expression in the hippocampus in Western blot. CUMS induced a higher level of NF-*κ*B p65 expression in C57BL/6 mice compared to control animals [*F* = 4.177, *p* = 0.032] (*p* < 0.05), while Tak242 relieved the alteration (*p* > 0.05, control group versus Tak242 group). CUMS induced an increase in NF-*κ*B p65 expression in ob/ob mice compared to control mice [*F* = 5.641, *p* = 0.015] (*p* < 0.01), while Tak242 could reverse the alteration (*p* < 0.05). Further, there were significant differences in NF-*κ*B p65 expression in the hippocampus between control groups (*t* = 5.548, *p* < 0.001), CUMS groups (*t* = 8.350, *p* < 0.001), and Tak242 groups (*t* = 4.592, *p* = 0.001) of C57BL/6 mice and ob/ob mice. The results indicated that ob/ob mice had a higher level of NF-*κ*B p65 in the hippocampus than C57BL/6 mice had; stress worsened the situation, while the antagonist normalized it.

#### 3.5.2. NF-*κ*B p65 Expression in the Frontal Cortex and Hippocampus in IHC

Figure 5(c) shows the NF-*κ*B p65 expression in the frontal cortex (above) and hippocampus (below) in immunohistochemistry (IHC). Figures 5(b) and 5(c) show the cell counting of NF-*κ*B p65 expression in the frontal cortex and hippocampus. The NF-*κ*B expression in IHC confirmed the change tendency of NF-*κ*B p65 expression in the frontal cortex and hippocampus in Western blot.

In Figure 5(d), CUMS induced significant NF-*κ*B p65 expression in the frontal cortex of C57BL/6 mice [*F* = 50.864, *p* < 0.001] (*p* < 0.01), while Tak242 reversed the change in mice of the Takc57 group (*p* < 0.01). CUMS induced an obvious NF-*κ*B p65 expression in the frontal cortex of ob/ob mice, but the differences in the number of NF-*κ*B p65-positive expression cells among the three groups did not reach significance [*F* = 2.275, *p* = 0.159]. There were no significant differences in the number of positive expression cells in the frontal cortex between C57BL/6 mice and ob/ob mice in control groups (*t* = 0.333, *p* = 0.750), CUMS groups (*t* = 0.323, *p* = 0.758), and Tak242 groups (*t* = 0.562, *p* = 0.595).

In Figure 5(e), CUMS induced a significant NF-*κ*B p65 expression in the hippocampus in the CUMSc57 group compared with the control group [*F* = 19.293, *p* < 0.001] (*p* < 0.01). Tak242 changed the alteration of NF-*κ*B p65-positive expression in the hippocampus in the Takc57 group (*p* < 0.01). CUMS induced a much more NF-*κ*B p65-positive expression in the hippocampus in the CUMSob group [*F* = 4.162, *p* = 0.053] (*p* < 0.05), while Tak242 relieved the increased NF-*κ*B p65 expression of the hippocampus in the Tak242 ob group (*p* > 0.05). ob/ob mice in the control group had significantly more positive expression cells than C57BL/6 mice had in the control group (*t* = 6.481, *p* = 0.001); no significant differences in the number of positive expression cells were found between C57BL/6 mice and ob/ob mice in the CUMS group (*t* = 2.114, *p* = 0.079) and Tak242 group (*t* = 1.759, *p* = 0.129).

## 4. Discussion

Studies have confirmed that stress was associated with elevated inflammation and depression, and increased cytokines in the brain played a crucial role in the etiology of depression [1]. Meanwhile, a low-grade inflammation state might constitute vulnerability to depression in an obese population [16] because the higher levels of certain cytokines in the prefrontal cortex and hippocampus were regarded as contributors of emotional disorder and memory damage [7]. The present study probed the depressive-like behaviors induced by CUMS between normal-body weight mice and ob/ob mice [37, 38] and the protective effects of the TLR4 antagonist on behavioral changes [22, 27, 28]. The results demonstrated that CUMS induced lower sucrose consumption, more time spent in the central squares, low number of movements in OFT, and less target quadrant time in MWM in both C57BL/6 mice and ob/ob mice. The results all suggested that the stressed animals displayed anhedonia and disinterest in a new environment and were in a state of low excitement and memory impairment [21, 22]. Inconsistent with the hypotheses, TLR4 antagonist Tak242 displayed different effects on preventing the behavioral disorders by showing that Tak242 remarkably reversed lower sucrose consumption and induced behavioral disorders in OFT and memory impairment except anhedonia in C57BL/6 mice and memory impairment in ob/ob mice. The findings implicated that overexpression of cytokines in the frontal cortex and hippocampus could not interpret all depressive symptoms; dysfunction of the hypothalamic-pituitary-adrenal axis (HPA) and the neurotransmitter system played important roles in the pathogenesis of depression [39, 40]. However, it was noticed that ob/ob mice in both control group and CUMS group displayed serious anhedonia and autonomic activity suppression than did C57BL/6 mice which suggested that obese animals might be more vulnerable to depression than normal weight animals under stress [41, 42].

Studies showed that certain cytokines such as IL-1*β* were involved in the pathogeneses of depression [2, 3]. TNF-*α* was closely related with locomotor activity suppression, anhedonia, and fatigue, as well as alterations in cognition, and might cause depressive symptoms through the HPA axis [40]. Our previous study also confirmed that the reduction level of IL-1*β*, IL-6, and TNF-*α* in the hippocampus could relieve depressive-like behaviors [5, 17]. In the study, CUMS increased the IL-1*β* and IL-6 expression in both C57BL/6 and ob/ob mice, while Tak242 prevented the alterations. The results supported the conclusion that stress elevated the inflammation condition and induced depression. Moreover, the present study showed that ob/ob mice in unstressed, stressed, and antagonist groups had higher levels of IL-1*β*, IL-6, and TNF-*α* in both frontal cortex and hippocampus than C57BL/6 mice which indicated that ob/ob mice had higher baseline of cytokines in certain brain areas; stress worsened the situation, while the TLR4 antagonist could not completely reverse the increased cytokines in ob/ob mice. Combined with the results that both unstressed and stressed ob/ob mice had serious depressive-like behaviors than C57BL/6 mice had, the study reached the conclusion that higher levels of cytokines of the frontal cortex and hippocampus might be the reason for the susceptibility to depression in obese mice [20].

TLR4-mediating innate immune response was associated with neuroinflammation [23, 24]. NF-*κ*B was a cardinal transcriptional regulator of inflammation and apoptosis of neurons [1, 10]. DAMPs activated the TLR4-NF-*κ*B pathway and induced elevated levels of IL-1*β*, IL-6, and TNF-*α* [16]. In order to clarify the role of TLR4-NF-*κ*B signaling in the regulation of inflammatory responses during CUMS, we examined the NF-*κ*B expression in the frontal cortex and hippocampus which are closely related with cognition and emotion using Western blot and immunohistochemistry. In the results of Western blot, CUMS induced a significant increase in NF-*κ*B expression in the frontal cortex and the TLR4 antagonist reversed the overexpression of NF-*κ*B in C57BL/6 mice. However, both CUMS and antagonist did not change the NF-*κ*B expression in the frontal cortex in ob/ob mice. This explains that ob/ob mice in the control group had high NF-*κ*B expression at baseline than C57BL/6 mice had, and CUMS failed to increase the level of NF-*κ*B in the frontal cortex. In addition, the present study shows that CUMS induced a significant increase in NF-*κ*B expression in the hippocampus while the TLR4 antagonist could reverse the overexpression of NF-*κ*B in both C57BL/6 mice and ob/ob mice, but all ob/ob mice displayed higher NF-*κ*B levels in the hippocampus than C57BL/6 mice did. The results suggested that the high NF-*κ*B expression at baseline was the crucial reason for the overexpression of cytokines in the hippocampus; CUMS stimulated higher levels of NF-*κ*B, but the antagonist could normalize in ob/ob mice. In the IHC result, CUMS induced NF-*κ*B overexpression in the frontal cortex and hippocampus in both C57BL/6 and ob/ob mice, and ob/ob mice in the control group had more obvious NF-*κ*B expression in the hippocampus than C57BL/6 mice had, which was consistent with the results in Western blot. Based on the findings, the study concluded that ob/ob mice had a hyperactive TLR4-NF-*κ*B signal and higher level of cytokines under CUMS [1, 21]. Our study may provide novel insights into the development of new therapeutic approaches for obesity comorbidity depression [39, 43].

## 5. Conclusions

Our results demonstrated that CUMS induced a depressive-like behavior, spatial memory damage, higher level of serum corticosterone, and overexpression of cytokines and NF-*κ*B in the frontal cortex and hippocampus. Obese mice displayed serious depressive-like behaviors and an upregulated TLR4-NF-*κ*B signal. The implications for understanding the pathoetiology of vulnerability to depression of obesity and the development of new treatments are also considered.

## Abbreviations

IL: Interleukin
TNF-*α*: Tumor necrosis factor-*α*
TLR4: Toll-like receptor 4
NF-*κ*B: Nuclear factor kappa B
CUMS: Chronic unpredictable mild stress
HPA: Hypothalamic-pituitary-adrenal
PFC: Prefrontal cortex
HIP: Hippocampus
ANOVA: Analysis of variance
SEM: Standard error of the mean.
